# Deep geological disposal of plastic waste: Pros and cons

**DOI:** 10.12688/f1000research.149702.1

**Published:** 2024-05-17

**Authors:** Hayk Minasyan

**Affiliations:** 1 Faculty of Biology, Yerevan State University, Yerevan, Yerevan, Armenia

**Keywords:** Plastic, waste, waste management, geological disposal.

## Abstract

Plastic production is growing, plastic waste is accumulating, and current waste management methods (recycling, incineration, etc.) are not yet able to solve this waste problem. Proposals and projects aimed at solving or facilitating the problem of plastic waste are therefore relevant.

This article proposes a deep geological disposal method for plastic waste projects. The prototype for this project was the deep geological disposal of nuclear waste. Although plastic waste and nuclear waste are fundamentally different, the adequacy of this approach is justified by the fact that plastic waste, such as nuclear waste, has a a long degradation period and poses great danger to the environment, animals, and humans. The article is conceptual and examines the aspects necessary for the implementation of the project, such as the establishment of a special international UN agency for plastic waste management, specific sources of funding, giving plastic waste a monetary value, applying free market principles, and using existing potential opportunities for deep geological disposal of plastic waste. This article also discusses the proposed concept for finding more optimal options.

## Introduction

Over the past 70 years, more than 10 billion tons of plastic have been produced. Approximately 10% of waste has been recycled, 11% burned, 8% buried, and the remaining 70% pollutes the environment ( Enfrin et al., 2019, Zaman and Newman, 2021). Approximately 80% of ocean plastic litter comes from land. More than 10 million tons of plastics enter the oceans annually ( Carney Almroth and Eggert, 2019, Chassignet et al., 2021). It is not known exactly how much plastic in the ocean remains on the surface, how much floats at different depths, or how much sinks to the bottom. The amount of macroplastics on the ocean surface is large. For example, it forms The Great Pacific Garbage Patch, which quickly captures new water surfaces ( Egger et al., 2020).

Large amounts of plastics are buried in landfills ( Rahman et al., 2023). Currently, global annual plastic production is approximately 348 million tons ( Yu et al., 2022). Nearly 80% of the total plastic waste is accumulated in landfills/open dumps and in the natural environment ( Yadav et al., 2020). As landfills are relatively closely sealed reactors with complex biochemical reactions and physical changes, plastic waste buried in landfills is subjected to more severe environmental conditions such as leachate pH, high salinity, fluctuating temperature, gas generation, physical stress, and microbial degradation. All the above factors may lead to the fragmentation of plastics to microplastics (MPs), and small plastic debris can be carried out by the discharge of leachate ( Wojnowska-Baryła et al., 2022, Zhu et al., 2021). The ultimate fate of plastic in landfills is a major concern, particularly as there is no established method for determining whether plastic degrades, biodegrades, or is recalcitrant. The amount of plastic waste continues to increase progressively, and this waste is increasing the land and water surfaces ( Singh et al., 2023). The accumulation of mismanaged plastic waste (MPW) in the environment is a global concern ( Lebreton and Andrady, 2019). Therefore, along with the already practiced approaches (recycling, incineration, landfill, etc.), new approaches to plastic waste management are necessary. The aim of this article is to offer deep geological disposal of plastic waste as a potentially new approach.

## Methods

We used Google Scholar, ResearchGate, ScienceDirect, SciSpace, Scopus, PubMed, Consensus, and Core search engines to determine the availability of deep geological plastic waste disposal in the scientific literature. We used long-tail keywords such as “deep geological disposal plastic waste”, “deep geological disposal”, and “geological disposal plastic”, and short-tail keywords “geological disposal” and “plastic waste.”

## Results

A search of the top search engines (Google Scholar, ResearchGate, ScienceDirect, SciSpace, Scopus, PubMed, Consensus, and Core) using both long- and short-tail keywords revealed no articles dedicated to the deep geological disposal of plastic waste. Therefore, the idea of deep geological disposal of plastic waste has been presented for the first time in this article.

## Discussion

Plastics have been destroyed very slowly in nature. It can take tens to hundreds of years ( Quecholac-Piña et al., 2020, Mohanan et al., 2020, Ru et al., 2020). In this sense, the disintegration of plastic waste is similar to that of radioactive waste. However, the decay time of radioisotopes occurs over a wider time interval. For example, srontium-90 and cesium-137 have half-lives of approximately 30 years whereas plutonium- 239 has a half-life of 24,000 years ( Ahluwalia, 2019). Both nuclear technologies and plastic production have been unprecedented in the history of mankind. Interestingly, the mass production of plastics began simultaneously with the development of nuclear power plants. Both occurred after World War II (the Second World War). The mass production of plastics began in 1950. Two million metric tons of plastic was produced in that year ( Geyer, 2020). In 1954, the world’s first nuclear power station in Obninsk (in the Soviet Union) began to produce electricity; in 1956, the Calder Hall nuclear power plant in the United Kingdom began to operate; and in 1958, the Shippingport Atomic Power Station in the USA began to generate electricity ( Alam et al., 2019, Geyer et al., 2017). In the case of mass production of plastic and the proliferation of nuclear power plants, the problem of waste appeared, but they were solved in different ways. The atomic explosions in August 1945 showed the power of nuclear weapons and the danger of nuclear waste; from the very beginning of the operation of nuclear power plants, nuclear waste was collected, and deep geological disposal has been considered the best solution ( Strandberg and Andrén, 2009). Regarding plastic waste, nothing has been done for a long time, and the situation is now approaching critical. Being invisible, radiation poses a serious risk to humans and the environment, whereas plastic disintegration is a chemical risk to ecology, animals, and humans.

Although nuclear waste and plastic waste are distinct areas, there are some similarities in their management ( Armand et al., 2023, Neksumi et al., 2022, Subba Rao et al., 2022, Zalasiewicz et al., 2019):

1. Both nuclear and plastic waste management have long-term environmental impacts. Nuclear waste contains radioactive materials that have remained hazardous for thousands of years. Similarly, certain types of plastic wastes, such as single-use plastics, can persist in the environment for hundreds of years.
2. Both nuclear and plastic waste management require appropriate storage and disposal to minimize their impact. Nuclear waste must be stored in secure facilities to prevent the leakage or release of radioactive materials. Similarly, plastic waste must be managed to prevent it from entering waterways, harming wildlife, or breaking down into MPs that can contaminate ecosystems.
3. Both nuclear and plastic waste management face public concerns and opposition. Nuclear waste is a hazard owing to radiation and long-term storage. Similarly, plastic waste has garnered attention owing to its environmental consequences.
4. Both nuclear and plastic waste management require ongoing research and technological advancements for more effective and sustainable solutions. Efforts are underway to enhance nuclear waste storage, explore advanced reactor designs, and develop waste-reprocessing technologies. Similarly, progress in plastic waste management includes the development of recycling technologies, exploration of alternatives, and implementation of waste reduction strategies.

Despite the aforementioned similarities with plastic waste management processes, nuclear waste management poses distinct challenges owing to its radioactive nature and long-term risks. Plastic waste management, on the other hand, focuses on reducing pollution and minimizing the environmental impact of plastic waste. But the comparison of plastic waste with nuclear waste is adequate in the sense that it shows: 1. The seriousness of the problem of plastic waste, 2. The need for broad international cooperation in managing plastic waste, and 3. The need to create (outside or inside UNEP) an Agency for Plastic Waste Management, which would be part of the UN system; 4. The need to create funds to raise money to solve the problem of plastic waste; 5. There is a need to apply some criteria for nuclear waste management to solve the problem of plastic waste.

The International Atomic Energy Agency (IAEA), established in December 1957 by the United Nations ( von Mehren, 1959) may be a model for creating the International Plastic Waste Agency. However, on many specific issues, the IAEA and plastic waste agencies will differ. For example, IAEA’s main sources of funding are the Regular Budget Fund, the Technical Cooperation Fund and Extra budgetary Program Funds that are supplied by Member States and by other donors ( Getmansky, 2017). The sources of funding for Plastic Waste Agency Management (PWAM) include governments, foundations, corporations, international and local public organizations, individuals, other donors and taxes paid by plastic producers and plastic item manufacturers. Establishing a connection between the volumes of plastic produced, the income of plastic producers, and the money spent on plastic waste management will make it possible to more accurately determine the cost of plastic ecology.

Extrapolation from nuclear waste disposal to plastic waste management may be useful. As mentioned above, deep geological disposal is considered the best solution for nuclear waste management ( Strandberg and Andrén, 2009). However, for deep geological disposal of plastic waste, the latter must be collected in advance. Nuclear waste is kept in containers at nuclear power plants and nuclear weapons factories, or stored in burial grounds isolated from contact with the environment. In contrast, plastic waste pollutes the environment everywhere; almost all people come into contact with it and contribute to its formation. To solve the problem of plastic waste collection, it is necessary to provide monetary value for this waste. It is promising to create a market for plastic waste and apply all the mechanisms of the free market and trade to it, especially because plastic waste can become a valuable primary raw material in the future.

With stable financing of the demand for plastic waste, firms, companies, and private enterprises engaged in the collection, transportation, and storage of plastic waste will spontaneously appear, and plastic producers themselves may want to create subsidiaries involved in the collection, transportation, and disposal of plastic waste. If plastic waste prices are set to make collecting and selling waste profitable, there could even be a fleet devoted solely to cleaning up plastic waste in the oceans. Ships specializing in the removal of debris from water basins can more successfully apply technologies that remove plastic without harming aquatic life.

If plastic waste is removed from the seas and oceans and collected from landfills, the question becomes what to do with all this, given that plastic waste cannot be recycled or burned. One possible solution could be to deposit plastic waste inside numerous exhausted, abandoned, and unused mines, quarries, caverns, and holes. This offer is supported by the following:

1. There are a large number of exhausted, abandoned, and unused mines, quarries, caverns, and holes in the world, and they themselves represent a major environmental problem ( Bennett, 2016, Cui et al., 2020, Kushwaha et al., 2019, Liu et al., 2021).
2. Abandoned mines, holes, and caverns often have existing infrastructure such as roads, railways, and buildings that can potentially be repurposed or reused ( Lele et al., 2023, Collier and Ireland, 2018). Many mines have been developed with significant infrastructure to support mining operations, including transportation systems and structures for housing workers or storing equipment ( Limpitlaw and Briel, 2014, Carvalho, 2017). If these abandoned sites are considered for redevelopment or re purposing, existing infrastructure can provide a foundation for future use. For example, roads and railways can be repaired or upgraded to facilitate transportation of plastic waste to the site. Buildings can be renovated or repurposed for various functions, such as offices, storage facilities, processing facilities, warehouses, research facilities, and even recreational spaces for workers. Buildings that were once used as housing for miners can still be structurally sound and repurposed as housing for new workers.
3. The use of abandoned mines and caverns as storage for oil, gas, or other minerals ( Du et al., 2022, Luo et al., 2022, Saigustia and Robak, 2021) has shown the possibility of their conversion to universal storage. It is especially noteworthy that abandoned mines have been used, are being used and will be used as repositories for nuclear waste ( Kasperski and Storm, 2020, Xie et al., 2020, Yim, 2022). Nations that employ nuclear power generally prefer to store radioactive waste by placing it in underground mines made up of a network of tunnels or passages linked to disposal tunnels situated several hundred meters below the surface ( Popov et al., 2019). However, considering abandoned mines and caverns as storage sites, it is important to assess the safety and stability of these spaces before any large-scale redevelopment or repurposing efforts. There are several important factors that make this option potentially problematic but not impossible.
1. Environmental impact: The placement of plastic waste in such locations can have significant environmental consequences. Plastic waste can release harmful chemicals over time, contaminating the soil, groundwater, and surrounding ecosystems. Pollution can have long-lasting effects on plants, animals, and human health.
2. Leachate concerns: Plastic waste can generate leachate, a liquid that forms when water percolates through waste. Leachates can contain toxic substances that can seep into the ground and contaminate groundwater sources, affecting drinking water supplies and further exacerbating environmental issues.
3. Potential for migration: Plastics are lightweight and can be easily carried by wind or water in the case of superficial disposal, potentially escaping from designated areas and spreading across the surrounding environment. This can lead to littering and pollution of nearby land, rivers, and oceans, exacerbating the global plastic waste problem rather than solving it.
4. Future land use: Some abandoned locations may have potential for future use, such as reclamation, habitat restoration, or even tourism. Using them as plastic waste dumping sites limits these possibilities and hinders the long-term sustainable development of these areas.

The above arguments for and against the use of abandoned mines, holes, and caverns for plastic waste disposal indicate that the idea of plastic waste disposal is not universal, and in each case, its application must be weighed and all local circumstances taken into account.

There is no data on how much plastic waste can be deposited in them, however, based on the depth and diameter of many quarries, it can be assumed that all plastic trash will fit in them for many decades and more to come. The deposition of waste should be carried out in accordance with the rules of conservation, with the possibility of extracting plastic waste in the event of depletion of hydrocarbon reserves on the planet ( Pang et al., 2022, Petrescu, 2020). Countries where mines and quarries are used to deposit plastic waste can profit from long-term leasing and storage of their mines and quarries. Parallel to depositing plastic waste in these locations, it is also indispensable to continue focusing on reducing the generation of plastic waste, promoting recycling and reuse, and developing sustainable waste management systems. Equally important is investment in proper waste infrastructure and raising awareness of plastic pollution. Implementing effective policies and regulations can help address the plastic waste crisis in an environmentally friendly and sustainable manner. Encouraging the development and use of non-biodegradable but environmentally friendly alternatives to plastics can also contribute to a long-term solution.

Thus, as soon as plastic waste becomes a commodity and an appropriate commercial approach, adequate pricing, and geological deposits are applied to it, the problem of plastic waste will be facilitated on the way to resolution.

## Conclusion

Conventional plastics are made from petroleum and natural gas. The latter are extracted from the bowels of Earth. According to the main concept of the article, extracted petroleum and natural gas can be partially returned to the bowels of the earth in the form of plastic waste that pollutes the environment and is currently not subject to recycling, reuse, repurposing, incineration, or other plastic waste management technologies. In the future, if petroleum and gas reserves on the planet are depleted, plastic waste at Earth’s depths can become a valuable raw material for the production of plastic and other hydrocarbon products. This approach to plastic waste management requires future research and discussion regarding the optimization of the concept, its practices, regulations, and policies.

## Data Availability

No data are associated with this article.
