# Deep geological disposal of plastic waste: Pros and cons

**DOI:** 10.12688/f1000research.149702.2

**Published:** 2025-07-17

**Authors:** Hayk Minasyan

**Affiliations:** 1 Faculty of Biology, Yerevan State University, Yerevan, Yerevan, Armenia

**Keywords:** Plastic, waste, waste management, geological disposal.

## Abstract

Plastic waste accumulation is a global environmental issue. Current methods (recycling, incineration, landfilling, etc.) are not a sustainable long-term solution and so far they cannot prevent continuous accumulation of plastic waste worldwide. This article introduces the concept of deep geological disposal (DGD) of plastic waste as an alternative strategy. The concept principally differs from the traditional landfilling. In the latter, plastic waste is affected by chemical, physical, and microbial factors that cause plastic fragmentation and environmental leakage. On the contrary, DGD isolates plastic waste in abandoned mines, quarries and caves, ensuring safe long-term containment of the waste and the possibility of its reuse as a raw material in the future. Another advantage of DGD is that these geological structures usually have transport infrastructure and storage facilities, and, as a result, they offer a more controlled environment with reduced risk of leachate, microplastic dispersion, and surface pollution. The paper discusses the practical, economic, and environmental aspects of the concept of DGD of plastic waste. The proposal does not offer to replace other available mechanisms of plastic waste management, it presents DGD as a complementary and potentially effective method for addressing non-recyclable and mismanaged plastic waste worldwide.

## Introduction

Over the past 70 years, more than 10 billion tons of plastics have been produced ( Geyer et al., 2017). A 2025 study (based on 2022 data) found that **27.9%** was recycled, **34%** was incinerated, **40%** went to landfills, and **11%** was *mismanaged* (i.e. littered or dumped into the environment) ( Houssini et al., 2025). Approximately 80% of ocean plastic litter comes from land. More than 10 million tons of plastics enter the oceans annually ( Carney Almroth and Eggert, 2019, Chassignet et al., 2021). There are more than 170 trillion plastic particles floating in the oceans ( Eriksen et al., 2023). The majority of plastic mass (90-98%) is contained in the large plastic items (>25 mm). Microplastics (<5 mm) and plastics between 5 and 25 mm form the small remainder ( Kaandorp et al., 2023).

Currently, global annual plastic production is approximately 348 million tons ( Yu et al., 2022). Nearly 80% of the total plastic waste is accumulated in landfills/open dumps and in the natural environment ( Yadav et al., 2020). As landfills are relatively closely sealed reactors with complex biochemical reactions and physical changes, plastic waste buried in landfills is subjected to more severe environmental conditions such as leachate, high salinity, fluctuating temperature, gas generation, physical stress, and microbial degradation. All the above factors may lead to the fragmentation of plastics to microplastics (MPs), and small plastic debris can be carried out by the discharge of leachate ( Wojnowska-Baryła et al., 2022). The ultimate fate of plastic in landfills is a major concern, particularly as there is no established method for determining whether plastic degrades, biodegrades, or is recalcitrant. The amount of plastic waste continues to increase progressively, and this waste is increasing the land and water surfaces ( Singh et al., 2023). The accumulation of mismanaged plastic waste (MPW) in the environment is a global concern ( Lebreton and Andrady, 2019). Therefore, along with the already practiced approaches (recycling, incineration, landfill, etc.), new approaches to plastic waste management are necessary. This paper proposes a new concept: deep geological disposal (DGD) of plastic waste as a long-term strategy. It has many advantages over landfills. Unlike landfills, DGD provides deep isolation and sealing of plastic waste. This prevents degradation and fragmentation of plastic under the influence of environmental factors and eliminates environmental pollution. In contrast to landfills, where plastic waste is buried (disposed of ) together with other municipal solid waste (MSW), the DGD involves disposing only selective plastic waste. This waste, currently unsuitable for recycling, may tomorrow become a raw material for future technologies. Conceptually, this waste is rethought not just as waste, but as a potential material for future use. In this way, DGD transforms the problem of plastic pollution from an intractable environmental problem to the storage of hydrocarbons as the feedstock of the future. This article outlines the rationale, feasibility and implications of this unconventional but potentially transformative strategy.

## Methods

We used Google Scholar, ResearchGate, ScienceDirect, SciSpace, Scopus, PubMed, Consensus, and Core search engines to determine the availability of deep geological plastic waste disposal in the scientific literature. We used long-tail keywords such as “deep geological disposal plastic waste”, “deep geological disposal”, and “geological disposal plastic”, and short-tail keywords “geological disposal” and “plastic waste.”

## Results

search of the top search engines (Google Scholar, ResearchGate, ScienceDirect, SciSpace, Scopus, PubMed, Consensus, and Core) using both long- and short-tail keywords revealed no articles dedicated to the deep geological disposal of plastic waste. Therefore, the idea of deep geological disposal of plastic waste has been presented for the first time in this article.

## Discussion

Plastic decomposes very slowly: it can take tens to hundreds of years ( Quecholac-Pina et al., 2020, Mohanan et al., 2020, Ru et al., 2020). In this sense, the disintegration of plastic waste is similar to that of radioactive waste. However, the decay time of radioisotopes occurs over a wider time interval. For example, srontium-90 and cesium-137 have half-lives of approximately 30 years whereas plutonium- 239 has a half-life of 24,000 years ( Ahluwalia, 2019). Both nuclear technologies and plastic production have been unprecedented in the history of mankind. Interestingly, the mass production of plastics began simultaneously with the development of nuclear power plants: both occurred after World War II (the Second World War). The mass production of plastics began in 1950. Two million metric tons of plastic was produced in that year ( Geyer, 2020). In 1954, the world's first nuclear power station began to produce electricity ( Alam et al., 2019, Geyer et al., 2017). In the case of mass production of plastic and the proliferation of nuclear power plants, the problem of waste appeared, but they were solved in different ways. The atomic explosions in August 1945 showed the power of nuclear weapons and the danger of nuclear waste; from the very beginning of the operation of nuclear power plants, nuclear waste was collected, and deep geological disposal has been considered the best solution ( Strandberg and Andrén, 2009). Regarding plastic waste, nothing has been done for a long time, and the situation is now approaching critical. Being invisible, radiation poses a serious risk to humans and the environment, whereas plastic disintegration is a chemical risk to ecology, animals, and humans.

Although nuclear waste and plastic waste are distinct areas, there are some similarities in their management ( Armand et al., 2023, Neksumi et al., 2022, Subba Rao et al., 2022, Zalasiewicz et al., 2019):

1. Both nuclear and plastic waste has long-term environmental impacts. Nuclear waste contains radioactive materials that have remained hazardous for thousands of years. Similarly, certain types of plastic wastes, such as single-use plastics, can persist in the environment for hundreds of years.
2. Both nuclear and plastic waste requires appropriate storage and disposal to minimize their impact. Nuclear waste must be stored in secure facilities to prevent the leakage or release of radioactive materials. Similarly, plastic waste must be managed to prevent it from entering waterways, harming wildlife, or breaking down that can contaminate ecosystems.
3. Both nuclear and plastic waste management face public concerns and opposition. Nuclear waste is a hazard owing to radiation and long-term storage. Similarly, plastic waste has garnered attention owing to its environmental consequences.
4. Both nuclear and plastic waste management require ongoing research and technological advancements for more effective and sustainable solutions.

Despite the aforementioned similarities with plastic waste management processes, nuclear waste management poses distinct challenges owing to its radioactive nature and long-term risks. Plastic waste management, on the other hand, focuses on reducing pollution and minimizing the environmental impact of plastic waste.

Extrapolation from nuclear waste disposal to plastic waste management may be useful. As mentioned above, deep geological disposal is considered the best solution for nuclear waste management ( Strandberg and Andrén, 2009). However, for deep geological disposal of plastic waste, the latter must be collected in advance. One of the reasons why people pollute the environment so irresponsibly with plastic waste is that this waste has no monetary value. If it had any value, perhaps people would pollute less, and many would collect plastic waste and get paid for it. Monetary value for plastic waste will facilitate plastic waste collection. If plastic waste is collected from the seas, oceans, and landfills, the question becomes what to do with the waste that cannot be recycled or burned? According to DGD concept, this waste may be deposited inside exhausted, abandoned, and unused mines, quarries, caverns, and holes. This offer is supported by the following:

1. There are a large number of exhausted, abandoned, and unused mines, quarries, caverns, and holes in the world ( Bennett, 2016, Cui, 2020, Kushwaha et al., 2019, Liu, 2021).
2. Abandoned mines, holes, and caverns often have existing infrastructure such as roads, railways, and buildings that can potentially be repurposed or reused ( Lele et al., 2023, Collier and Ireland, 2018). Many mines have been developed with significant infrastructure to support mining operations, including transportation systems and structures for housing workers or storing equipment ( Limpitlaw and Briel, 2014, Carvalho, 2017). If these abandoned sites are considered for redevelopment or re purposing, existing infrastructure can provide a foundation for future use. For example, roads and railways can be repaired or upgraded to facilitate transportation of plastic waste to the site. Buildings can be renovated or repurposed for various functions, such as offices, storage facilities, processing facilities, warehouses, research facilities, and even recreational spaces for workers. Buildings that were once used as housing for miners can still be structurally sound and repurposed as housing for new workers.
3. The use of abandoned mines and caverns as storage for oil, gas, or other minerals ( Du et al., 2022, Liu and Pei, 2021, Luo et al., 2022, Saigustia and Robak, 2021) has shown the possibility of their conversion to universal storage. It is especially noteworthy that abandoned mines have been used, are being used and will be used as repositories for nuclear waste ( Kasperski and Storm, 2020, Xie et al., 2020, Yim, 2022).
4. The placement of plastic waste in such locations is safe and has no environmental consequences. Plastic waste can release harmful chemicals over time in landfills, contaminating the soil, groundwater, and surrounding ecosystems whereas placement of plastic waste in isolated and encapsulated conditions prevents plastic degradation.
5. Leachate concerns: In landfills plastic waste can generate leachate, a liquid that forms when water percolates through waste. Leachates can contain toxic substances that can seep into the ground and contaminate groundwater sources, affecting drinking water supplies and further exacerbating environmental issues. In DGD leachate generation is excluded because plastic is kept in dry condition without physical and chemical influence.
6. Potential for migration: In landfills plastics are lightweight and can be easily carried by wind or water in the case of superficial disposal, potentially escaping from designated areas and spreading across the surrounding environment. This can lead to littering and pollution of nearby land, rivers, and oceans, exacerbating the global plastic waste problem rather than solving it. In DGD plastic waste migration is excluded.

There is no data on how much plastic waste can be deposited in DGD, however, based on the depth and diameter of many quarries, it can be assumed that all plastic trash will fit in them for many decades and more to come. The deposition of waste should be carried out in accordance with the rules of conservation, with the possibility of extracting plastic waste in the event of depletion of hydrocarbon reserves on the planet ( Pang et al., 2022, Petrescu, 2020).

Thus, as soon as plastic waste becomes a commodity and an appropriate commercial approach, adequate pricing, and geological deposits are applied to it, the problem of plastic waste will be facilitated on the way to resolution.

## Conclusion

Conventional plastics are made from petroleum and natural gas. The latter are extracted from the bowels of Earth. According to the concept of DGD, extracted petroleum and natural gas can be partially returned to the bowels of the earth in the form of plastic waste that is currently not subject to recycling, reuse, repurposing, incineration, or other plastic waste management technologies. In the future, if petroleum and gas reserves on the planet are depleted, DGD plastic waste can become a raw material for recycling and secondary production of plastic and other hydrocarbon products.

## Data Availability

No data are associated with this article.
