# Supplementary material for: Deep geological disposal of plastic waste: Pros and cons
Source: F1000Res. 2026 Jan 14;13:504. Originally published 2024 May 17. [Version 3] doi: 10.12688/f1000research.149702.3 (PMC12865289; doi:10.12688/f1000research.149702.3)
Supplement: Peer review history

# Reviewer report: Reviewer response for version 3

**DOI:** 10.5256/f1000research.194890.r450146

**Published:** 2026-02-02

**Authors:** Atik Fayshal

**Affiliations:** 1 North Dakota State University (NDSU), Fargo, North Dakota, USA

Accept without any further revision

Is the topic of the opinion article discussed accurately in the context of the current literature?

Partly

Are arguments sufficiently supported by evidence from the published literature?

Yes

Are all factual statements correct and adequately supported by citations?

Partly

Are the conclusions drawn balanced and justified on the basis of the presented arguments?

Yes

Reviewer Expertise:

GIS, Hydrothermal Liquefaction, Environmental Photochemistry, Pesticides, Organic Micropollutants, Plastic Waste

I confirm that I have read this submission and believe that I have an appropriate level of expertise to confirm that it is of an acceptable scientific standard.

# Reviewer report: Reviewer response for version 2

**DOI:** 10.5256/f1000research.184634.r437500

**Published:** 2026-01-02

**Authors:** Atik Fayshal

**Affiliations:** 1 North Dakota State University (NDSU), Fargo, North Dakota, USA

- **Lack of Economic Feasibility**: The manuscript does not provide a detailed economic analysis of deep geological disposal (DGD), including the costs of transporting, storing, and maintaining plastic waste in geological sites. A more thorough cost-benefit analysis is essential to evaluate the viability of this solution.
- **Environmental Risk Assessment**: While the paper claims DGD is safe, it does not address potential long-term environmental risks, such as groundwater seepage or geological changes that could compromise the waste storage. A robust risk assessment and detailed mitigation strategies are needed.
- **Inconsistent Degradation of Plastics**: The manuscript presents plastic waste as both inert (resistant to degradation) and a potential source of harmful leachates. This inconsistency should be clarified. If plastics are inert, leachate formation shouldn't be a concern; if not, the types of degradation products (e.g., microplastics) should be discussed.
- **Overlooking the Scale of Plastic Waste**: While the manuscript compares plastic waste to nuclear waste, it fails to address the vastly larger volume and less hazardous nature of plastic. The logistics of managing such a large volume in deep geological sites are not fully explored, and the feasibility of this approach should be reassessed in the context of the scale of the problem.
- **Comparison with Other Waste Management Methods**: The manuscript mentions DGD as a solution but does not sufficiently compare it to other methods like recycling, incineration, or energy recovery. These alternatives, particularly waste-to-energy systems, could be more cost-effective and environmentally sustainable, and DGD should be presented as a complementary strategy.
- **Funding and Commercial Viability**: The paper lacks a clear funding strategy for DGD. How will the infrastructure for DGD be financed? There is no discussion of whether this solution would rely on public or private funding or the economic incentives for businesses to invest in such an infrastructure.
- **Regulatory and Policy Considerations**: The manuscript does not discuss the regulatory frameworks required for implementing DGD globally. How will governments, international bodies, and environmental agencies regulate and oversee the implementation of DGD? A policy discussion would add depth to the feasibility of the proposed solution.
- **Clarification of Plastic as a Resource**: The idea of treating plastic waste as a resource is promising, but the manuscript should explore how this can be realized. How will plastic waste be extracted and reused from geological storage in the future? Further explanation is needed on the potential future technologies that could make this process feasible.

Is the topic of the opinion article discussed accurately in the context of the current literature?

Partly

Are arguments sufficiently supported by evidence from the published literature?

Yes

Are all factual statements correct and adequately supported by citations?

Partly

Are the conclusions drawn balanced and justified on the basis of the presented arguments?

Yes

Reviewer Expertise:

GIS, Hydrothermal Liquefaction, Environmental Photochemistry, Pesticides, Organic Micropollutants, Plastic Waste

I confirm that I have read this submission and believe that I have an appropriate level of expertise to confirm that it is of an acceptable scientific standard, however I have significant reservations, as outlined above.

# Response

**Published:** 2026-01-06

**Authors:** Hayk Minasyan

**Affiliations:** Biology, Yerevan State University, Yerevan, Yerevan, Armenia

**Response to Reviewer**

 We would like to sincerely thank the reviewer for the careful reading of our manuscript and for the constructive and insightful comments. We appreciate the opportunity to improve the clarity, depth, and balance of the article. Below we address each point in detail. Where appropriate, corresponding clarifications and extensions have been incorporated into the revised manuscript.

**1. Lack of Economic Feasibility**

**Reviewer comment:**

*The manuscript does not provide a detailed economic analysis of deep geological disposal (DGD), including the costs of transporting, storing, and maintaining plastic waste in geological sites. A more thorough cost-benefit analysis is essential to evaluate the viability of this solution.*

**Response:**

 We thank the reviewer for this important observation. We fully agree that the long-term viability of any waste-management strategy requires a structured economic analysis. In the present work, our intention was primarily to introduce and conceptually frame deep geological disposal (DGD) for plastic waste, rather than to present it as a fully developed operational program. For this reason, we initially limited ourselves to general economic considerations.

 In the revised manuscript, we clarify the conceptual nature of the proposal and describe the key economic parameters that should be evaluated in future feasibility studies, including transport, storage infrastructure, monitoring, maintenance costs, and the avoided externalities associated with environmental leakage of mismanaged plastic waste. We also emphasize that DGD is intended particularly for plastic fractions that are currently non-recyclable and environmentally persistent.

**2. Environmental Risk Assessment**

**Reviewer comment:**

*While the paper claims DGD is safe, it does not address potential long-term environmental risks…*

**Response:**

 We are grateful for this observation. We agree that any long-term storage concept must account for geological stability, groundwater interaction, and low-probability but high-impact scenarios.

 In the revised manuscript, we expand the discussion of environmental risk. We clarify that the DGD concept is based on using stable geological formations comparable to those considered internationally for hazardous or radioactive waste repositories. We also describe generic mitigation principles such as multi-barrier containment, hydrogeological isolation, site-selection criteria, and monitoring. At the same time, we explicitly acknowledge that a full probabilistic risk assessment requires dedicated interdisciplinary study beyond the scope of this conceptual article.

**3. Inconsistent Degradation of Plastics**

**Reviewer comment:**

*The manuscript presents plastic waste as both inert and a potential source of leachates…*

**Response:**

 We appreciate this insightful clarification request. The seeming inconsistency arises from the fact that plastics are generally resistant to biodegradation but may undergo fragmentation or leaching under certain environmental conditions, particularly in landfills where temperature, moisture, and chemical exposure vary.

 We have revised the text to state more clearly that **leachate formation and fragmentation concern conventional landfill environments**, whereas **DGD is designed to isolate plastics in dry, sealed, and geologically stable formations** where such degradation processes are expected to be minimized. Thus, one of the main advantages of DGD is precisely the reduction of environmental exposure that promotes degradation and dispersion.

**4. Overlooking the Scale of Plastic Waste**

**Reviewer comment:**

*The manuscript does not fully address the scale of plastic waste compared with nuclear waste…*

**Response:**

 We thank the reviewer for raising this central point. Our comparison with nuclear waste was not intended to imply equivalence of risk, but rather to illustrate how geological isolation concepts may be transferred and adapted.

 In the revised manuscript, we more clearly emphasize the **difference in scale and hazard profile**. We also clarify that DGD is **not proposed as a universal solution for all plastic waste**, but as a targeted approach for **non-recyclable and mismanaged fractions**. We expand the discussion on logistical feasibility at regional or national levels rather than on a single global repository model.

**5. Comparison with Other Waste-Management Methods**

**Reviewer comment:**

*The manuscript should better compare DGD with recycling, incineration, and energy recovery…*

**Response:**

 We fully agree, and we appreciate the reviewer’s suggestion. In the revised version, we expand the discussion to present DGD explicitly as a **complementary component of an integrated waste-management system**. We note that recycling, chemical recycling, incineration, and waste-to-energy technologies remain essential, and DGD is proposed specifically for streams that remain outside these systems due to technical or economic barriers.

**6. Funding and Commercial Viability**

**Reviewer comment:**

*The paper lacks a clear funding strategy…*

**Response:**

 We thank the reviewer for pointing out this important issue. As the paper is primarily conceptual, we did not originally propose specific financial frameworks. However, we fully agree that economic governance will be critical.

 We have now added a section discussing **potential funding pathways**, including extended producer responsibility schemes, public–private partnerships, environmental funds, and incentives linked to assigning economic value to currently mismanaged plastic waste. We also stress that financing models would require coordination between governments, industry, and international organizations.

**7. Regulatory and Policy Considerations**

**Reviewer comment:**

*The manuscript does not discuss the regulatory frameworks required for DGD…*

**Response:**

 We appreciate this observation. Implementation of DGD would indeed require robust regulatory oversight. In the revised manuscript, we discuss the need for:

 • national and international regulatory alignment

 • repository licensing and monitoring obligations

 • environmental impact assessment requirements

 • public-engagement mechanisms

 We also note that existing frameworks for hazardous-waste or nuclear-waste management provide precedents that may guide the evolution of DGD governance.

**8. Clarification of Plastic as a Resource**

**Reviewer comment:**

*Further explanation is needed regarding future reuse of stored plastic…*

**Response:**

 We thank the reviewer for highlighting this conceptual pillar. We now expand the manuscript to describe how **future chemical recycling, depolymerization, or hydrocarbon-recovery technologies** may enable retrieval and reuse of stored plastic waste.

 We clarify that **DGD is conceptualized not as irreversible burial**, but as **secure long-term storage** that preserves the material’s future value while eliminating environmental leakage today.

**Closing Statement**

 We are sincerely grateful to the reviewer for the depth and constructiveness of the comments. We believe that the revisions made in response have strengthened the manuscript significantly by improving conceptual clarity, balance, and feasibility framing.

# Reviewer report: Reviewer response for version 2

**DOI:** 10.5256/f1000research.184634.r432879

**Published:** 2026-01-02

**Authors:** Dr. Melanie Bergmann

**Affiliations:** 1 Alfred Wegener Institute, Helmholtz Centre for Polar and Marine Research, Bremerhaven, Germany

Review of Deep geological disposal of plastic waste: Pros and cons

 The opinion article by Hayk Minasyan deals with the long-term disposal of plastic waste and proposes disposal in the deep geosphere as a possible complimentary solution to plastic waste management.

 As this is an opinion piece, I should state that I disagree with the suggested concept, because, it is not in line with the waste hierarchy. Secondly, I very much doubt that this approach is feasible economically speaking and in terms of available capacity, or unproblematic for the environment in the long term. I don't mean to be polemical, but this idea is a little akin to proposing to shoot our plastic waste into space. I am sorry but I recommend rejection on the following more specific grounds:

 No data is provided to show that this is a good idea.

 -Scalability: According to the OECD, plastic waste is due to triple by 2060 if business-as-usual growth rates of plastic production will continue on the current trajectory. This would cause 1014.144 million t of plastic waste in 2060 alone. No data are provided on the potential global storage capacity of DGD and how this relates to the projected mass of plastic waste. I suspect it could only accommodate a small fraction ever of the waste produced.

 -Implementation: Before deep storage, plastic waste would need to be collected. However, waste collection is currently poorly developed in many low-GDP countries. Development of new schemes would incur high cost, as would transport to these. It is unclear how new or improved waste collection infrastructures could be financed. In light of this, it seems unlikely that DGS could play a significant role.

 -Potential effects of hazards on geosphere integrity: Deep geological storage could be affected by the effects of climate change or other unforeseen changes in the geosphere (s. below example of Asse II mine for nuclear waste). Climate change does not only affect the geosphere of permafrost but also seismic activities increasing the frequency of earthquakes. This is a result of changes in hydrostatic pressure in the underground due to global sea level rise and more frequent strong storms, a recent study has shown. It could lead “to changes in seismic cycles – with an increasing risk of earthquakes and cascading effects such as landslides, tsunamis and liquefaction, particularly in the world's coastal regions.” Surely, such risks have to be analysed and considered in an era of climate change. Hazard assessments are needed and might show that only few areas exist that are sufficiently stable and thus amenable to deep geological storage. This aspect is not mentioned at all in the Opinion Piece.

 In addition, I would argue that those areas with greater resilience should be prioritised for the disposal of nuclear waste, given that it is much more hazardous than plastic waste, particularly during the acute phase. This leads me to another weak point in the argument: the comparison between the two types of waste. The text suggests that deep geological disposal is justified because plastic is persistent and toxic too. While this is true to a certain extent, nuclear waste is far more hazardous, making this comparison questionable. Furthermore, I do not consider the amount of text devoted to radioactive waste and its development to be relevant in an article about plastic waste.

 -Competition with other uses: Deep geological storage is considered for various purposes, including- as stated by the authors- nuclear waste as well as carbon that has been captured from the atmosphere to reduce CO2.  Competition between these uses and limited storage capacity raises again the question of scalability.

 More specific points:

 Abstract:

 “ *Approximately 80% of ocean plastic litter comes from land.*” This is a myth there is no clear source of this statement that is being re-iterated. By contrast, many studies find other percentages. Either omit this sentence or write that there is uncertainty attached to this estimate.

 Introduction

 -“ *More than 10 million tons of plastics enter the oceans annually (Carney Almroth and Eggert, 2019, Chassignet et al., 2021).”* This is outdated and the citation is not a data source (should be Jambeck et al. 2015). Borrelle et al. (2020) report that 19-23 million t/year enter the aquatic environment. Cottom et al. (2024) estimate plastic waste emissions at 52.1 [48.3–56.3] million metric tonnes (Mt) per year (all environments).

 -“ *A 2025 study (based on 2022 data) found that 27.9% was recycled, 34% was incinerated, 40% went to landfills, and 11% was mismanaged (i.e. littered or dumped into the environment) (Houssini et al., 2025)*.” Houssini et al. referred to recycling rates of 9% : “ *The global recycling rate remain stagnant showing little progress from previous estimates* *^1^* *, accounting of 9% of primary production*.” Please, correct or refer to the OECD report, which many people cite: “After taking into account losses during recycling, only 9% of plastic waste was ultimately recycled, while 19% was incinerated and almost 50% went to sanitary landfills. The remaining 22% was disposed of in uncontrolled dumpsites, burned in open pits or leaked into the environment.” file:///C:/Users/bergmann/Downloads/de747aef-en.pdf)

 -Maybe you want to add in the first paragraph that numbers will be much bigger if nanoplastics are added based on this study: (Refer to ref no.1)

 -“ *Currently, global annual plastic production is approximately 348 million tons (Yu et al., 2022)*.”

 This is outdated. OECD reports 460 million t already in 2019, which was 6 years ago, during which additional plastic has been produced at increasing growth rates. Some say, if plastic fibers are included, it should be ~500 million t in 2021.

 -“ *The accumulation of mismanaged plastic waste (MPW) in the environment is a global concern ( Lebreton and Andrady, 2019* *). Therefore, along with the already practiced approaches (recycling, incineration, landfill, etc.), new approaches to plastic waste management are necessary.“*

 This list of measures does not include reducing plastic production, even though this is one of the most effective ways to reduce plastic waste and plastic pollution (e.g. Lau et al. 2020; and hence the waste hierarchy), especially since it has recently been shown that plastic production and plastic pollution are linked (Cowger et al. 2024).

 -“ *As landfills are relatively closely sealed reactors with complex biochemical reactions and physical changes, plastic waste buried in landfills is subjected to more severe environmental conditions such as leachate, high salinity, fluctuating temperature, gas generation, physical stress, and microbial degradation*.” Please add storms and extreme weather events as well as natural disasters to this list.

 -P3: “ *The amount of plastic waste continues to increase progressively, and this waste is increasing the land and water surfaces (Singh et al., 2023)*.” Add ‘on’

 -“This waste, currently unsuitable for recycling, may tomorrow become a raw material for future technologies.” Please, elaborate, what might be these future uses? I am not aware of any such development and am skeptical as ‘plastics’ comprise so many different polymer types and at least 16,000 chemicals, so the potential combination of substances is huge, making any use unlikely. Just pointing to some potential future use is not sufficient in a scientific perspective, it is a bit like a unicorn, wishful thinking. This is important, because if there will be no such solution available, it is just another form of storage, and storage capacity is limited (especially given the expected growth of waste).

 -“ *This article outlines the rationale, feasibility and implications of this unconventional but potentially transformative strategy.*” I would argue that this article does not outline the feasibility of Deep Geological Disposal as no data are provided on the storage capacity in relation to waste generation.

 Method / Results

 -Method & Results seem appropriate for the limited scope intended. It only shows that this concept has not already been proposed elsewhere. Unfortunately, however, it would have been more important to provide data on the storage capacity in relation to waste generation to be able to assess the feasibility of the concept as stated above. Since this is lacking, I consider the methods/results insufficient.

 Discussion

 -P.4: *srontium-90* should be strontium-90

 -Even if decay times of plastic and nuclear waste are similar (we don’t actually know these for many plastics), I find the analogy made and thus the framing misleading, given that nuclear waste is so much more hazardous.

 -“ *One of the reasons why people pollute the environment so irresponsibly with plastic waste is that this waste has no monetary value. If it had any value, perhaps people would pollute less, and many would collect plastic waste and get paid for it*.“

 This sentence implies that plastic pollution is primarily driven by ‘irresponsible’ consumer behaviour. While this might be true in some cases, it has been shown that plastic pollution is linked with oversupply, i.e. increasing plastic production (Cowger et al.). This increase meets insufficient waste management capacity, so it is a structural problem, nothing that consumers can solve.

 -“1. *There are a large number of exhausted, abandoned, and unused mines, quarries, caverns, and holes in the world (* *Bennett, 2016* *, Cui, 2020* *, Kushwaha et al., 2019* *, Liu, 2021* *).”* This is insufficient as a proof of concept for DGD. Numbers plus capacity of such infrastructures mapped against plastic waste produced would be a starting point.

 -“ *4.The placement of plastic waste in such locations is safe and has no environmental consequences. Plastic waste can release harmful chemicals over time in landfills, contaminating the soil, groundwater, and surrounding ecosystems whereas placement of plastic waste in isolated and encapsulated conditions prevents plastic degradation.”* See argument above re potential geo- hazards in a changing climate.

 In addition, there is precedence for instability of former mines, e.g. the German salt mine Asse II, which is used to store radioactive waste ( https://www.bge.de/en/asse/ ). This raises the important question how isolated & safe such infrastructures really are in the long run (potentially even more so in a changing climate). At ASSE II, it is not even well-known what/how much was stored in the 1970’s. How can we ensure that future generations will have such knowledge, when we were unable to do so in the past in a ‘well-developed’ country such as Germany. There are likely more such examples elsewhere.

 -“ *The deposition of waste should be carried out in accordance with the rules of conservation, with the possibility of extracting plastic waste in the event of depletion of hydrocarbon reserves on the planet ( Pang et al., 2022, Petrescu, 2020).*” Once hydrocarbons will have been depleted, we will no longer be permitted to emit CO2 (even from incinerating DGD plastic waste), and hopefully long before that, in order to protect our climate and livelihoods. Has the author not heard of the climate protection agreement?

 -Should the paper still be accepted, I recommend review by geologists with a deep knowledge of the deep geosphere and its stability.

Is the topic of the opinion article discussed accurately in the context of the current literature?

Partly

Are arguments sufficiently supported by evidence from the published literature?

No

Are all factual statements correct and adequately supported by citations?

Yes

Are the conclusions drawn balanced and justified on the basis of the presented arguments?

No

Reviewer Expertise:

Plastic pollution

I confirm that I have read this submission and believe that I have an appropriate level of expertise to state that I do not consider it to be of an acceptable scientific standard, for reasons outlined above.

## References

[1] : Nanoplastic concentrations across the North Atlantic. *Nature* .2025;643(8071) : 10.1038/s41586-025-09218-1 412-416 10.1038/s41586-025-09218-1 40634739 PMC12240857

# Response

**Published:** 2026-01-02

**Authors:** Hayk Minasyan

**Affiliations:** Biology, Yerevan State University, Yerevan, Yerevan, Armenia

**Response to Reviewer:**

 We would like to  thank the Reviewer for the careful, detailed and critical evaluation of our Opinion Article and for the opportunity to clarify several points. We acknowledge that the topic of deep geological disposal (DGD) of plastic waste is unconventional and controversial. Precisely for this reason, we consider it important that it is openly discussed in the scientific literature.

 Below, we address the Reviewer's main concerns point-by-point.

**General position of the Reviewer:**

 The Reviewer states that they disagree with the concept because it is not aligned with the waste hierarchy, and that they doubt feasibility, scalability and long-term environmental safety.

 We fully respect this position. However, our article **does not propose DGD as a replacement for the waste hierarchy**, but as a **complementary, last-resort strategy** for those fractions of plastic waste that:

- cannot currently be recycled,
- are being persistently mismanaged,
- and contribute to long-term environmental loading.

This is clarified in several parts of the manuscript. The intention of the Opinion Article is **to explore whether DGD deserves consideration alongside existing measures** rather than to claim that it should become a dominant or primary strategy.

**1. “No data are provided to support that this is a good idea” / “Scalability and capacity are unclear”**

 We agree with the Reviewer that quantitative modelling of global storage potential versus projected plastic waste generation would be highly desirable. However, such work requires interdisciplinary collaboration between geologists, mining engineers, waste scientists and economists — and currently **no such models exist in the literature**.

 The aim of the Opinion Article is to **open this line of inquiry**, not to prematurely close it. We explicitly acknowledge that feasibility depends on:

- geological availability,
- storage integrity,
- cost and infrastructure,
- interaction with other societal priorities.

Therefore, the absence of full capacity models should be viewed not as a weakness of logic, but as an indication of **an emerging research direction that requires development**.

 We also emphasise that **DGD is not proposed for 100% of global plastic waste**, but potentially for *a limited fraction of persistent and unrecyclable waste*. Thus, its scalability must be evaluated in that narrower context.

**2. “Implementation challenges in low-income countries make DGD unrealistic”**

 We agree that insufficient waste collection remains a critical bottleneck globally. However, *any* advanced waste management strategy — including recycling and controlled incineration — depends on improving collection systems.

 Therefore, improving waste collection is not an argument *against* DGD specifically, but a **shared prerequisite across all management approaches**.

 Our intention is not to provide a financial blueprint, but to state that **if unrecyclable plastic must be isolated for centuries to millennia, deep stable formations may be one of few environments capable of achieving this**.

**3. “Geological instability and climate-related hazards may compromise DGD safety”**

 This is an important and valid consideration. We agree that:

- long-term hazard assessment,
- site-specific geological stability studies,
- and climate-sensitivity analyses

are indispensable for any future exploration of DGD.

 However, it should be noted that **geological disposal is already being developed internationally for high-level radioactive waste**, which requires far more stringent containment than plastics. This demonstrates that **stable deep formations do exist and can be evaluated rigorously**.

 We do not claim that all geological settings are suitable. Rather, we argue that **some subset of formations may meet the necessary stability criteria** — and that this deserves assessment.

**4. “Comparison with nuclear waste is misleading”**

 We agree that radioactive waste is vastly more hazardous. Our comparison **does not imply equivalence of risk**. The analogy is limited to **timescale**:

- both classes of material persist on century-to-millennium scales,
- both create cumulative environmental burden.

Thus, the analogy is conceptual, not risk-equivalence-based. We have clarified this in the revised text.

**5. “Competition for underground space (e.g., nuclear waste, CO** **₂** **storage)”**

 This is again a valid point — and another reason why **quantitative assessments are needed**. However, current planning for geological repositories is not expected to occupy all suitable formations globally. The Earth’s crust is vast, and different formations may be suited for different materials.

 We do not argue that DGD *must* proceed — only that **it should be considered among strategic options for long-term isolation of persistent wastes**.

**6. “Alleged ‘myth’ regarding land-based contribution to marine plastic pollution”**

 We acknowledge the Reviewer’s concern. We have revised the text to:

- reflect uncertainty ranges,
- avoid implying precision where none exists,
- and cite recent estimates alongside earlier work.

We agree that estimates differ and this is now clearly stated.

**7. “Recycling figures quoted incorrectly / outdated production data”**

 We thank the Reviewer for drawing attention to this. We have revised the manuscript to:

- ensure consistency with OECD and other authoritative sources,
- update global production estimates,
- correct the quoted recycling percentages.

We are grateful for this correction.

**8. “Future reuse of deposited plastic is speculative”**

 We accept this point. We have clarified that potential future retrieval is **hypothetical**, and that the primary argument for DGD remains **long-term isolation**, not resource banking.

**9. “Plastic pollution is linked to overproduction, not only consumer behaviour”**

 We fully agree. The revised text reflects that:

- structural production dynamics,
- insufficient waste management capacity,
- and global inequalities

are key drivers of environmental leakage.

**10. “Insufficient demonstration of feasibility in the Methods/Results”**

 We agree that the article does not supply engineering feasibility evidence — because it is not intended as an engineering design study. Instead, it is **a conceptual Opinion Article arguing that DGD deserves structured scientific evaluation rather than being dismissed a priori**.

 We are grateful to the Reviewer for their thoughtful engagement with our work. The primary purpose of this Opinion Article was not to present a finalized engineering solution, but rather to place a new idea into the scientific domain and initiate discussion — even if only among a limited readership. We also believe that the Review process itself illustrates an enduring feature of academic life: genuinely new ideas often encounter resistance, particularly when they challenge established hierarchies of thought. Such resistance is natural and even necessary — it sharpens arguments and clarifies assumptions. At the same time, it reinforces the importance of preserving intellectual space for unconventional perspectives, especially in areas where conventional approaches alone have not solved the problem.

 In summary, we remain convinced that the idea merits discussion, irrespective of whether one ultimately supports or rejects it. If the article has succeeded in stimulating such discussion, then it has fulfilled its intended purpose

# Reviewer report: Reviewer response for version 2

**DOI:** 10.5256/f1000research.184634.r398933

**Published:** 2025-07-29

**Authors:** Win Cowger

**Affiliations:** 1 The Moore Institute for Plastic Pollution Research, Long Beach, California, USA

I have reviewed the new manuscript and authors comments. I think the manuscript is greatly improved and am ok with moving to accept it. In general I have many fewer disagreements with the new manuscript and the ones that I have are more about my own personal opinions rather than the substance of the paper and arguments the author is making. I do recommend the author check the manuscript one more time as I did find a grammatical or formatting error "environment so irresponsibly with plastic васте is that this waste has".

Is the topic of the opinion article discussed accurately in the context of the current literature?

Partly

Are arguments sufficiently supported by evidence from the published literature?

Partly

Are all factual statements correct and adequately supported by citations?

Partly

Are the conclusions drawn balanced and justified on the basis of the presented arguments?

Partly

Reviewer Expertise:

Waste management, plastic pollution, watershed fate and transport

I confirm that I have read this submission and believe that I have an appropriate level of expertise to confirm that it is of an acceptable scientific standard.

# Response

**Published:** 2025-07-29

**Authors:** Hayk Minasyan

**Affiliations:** Biology, Yerevan State University, Yerevan, Yerevan, Armenia

Dear Dr. Win Cowger,

 Thank you very much for your feedback on the second version of my article. Your constructive and unbiased review of the first version has significantly improved the article. Your comments are especially valuable as you are a leading, recognized, and respected researcher and expert in the field.

 Thank you

 Sincerely

 Hayk Minasyan

 P.S. The error you pointed out is the result of incorrect formatting of the fonts of the word "waste". I have asked the editorial team of the journal to correct the error.

# Reviewer report: Reviewer response for version 1

**DOI:** 10.5256/f1000research.164194.r389475

**Published:** 2025-06-19

**Authors:** Win Cowger

**Affiliations:** 1 The Moore Institute for Plastic Pollution Research, Long Beach, California, USA

Summary:

 This is a commentary on the author's perspective about whether deep geological disposal of plastic makes sense and how it could be achieved. 

 Major Issues:

 - Abstract not standard format, split into one two sentence paragraph and one longer one. 

 - "70% of plastic is polluting the environment" seems high, papers cited are not primary literature for that number. I recommend looking into the original literature those numbers came from and determine if that is accurate and cite the original source. 

 - This sentence is confusing:  "For example, it forms The Great Pacific Garbage Patch, which quickly captures new water surfaces ( Egger et al., 2020)."

 - Many plastics in introduction just say there are large amounts of plastic, I think as a scientific study there should be numbers specifically stated, not just large amounts. For example, there are sources you can cite for how much plastic is in the ocean surface. I have added a few. 

 - The first two sentences in the discussion could be combined. 

 - Unclear in text what the author means by plastic disintegration, do they mean leachates, microplastics, nanoplastics?

 - Some of the first paragraph of the discussion seems unnecessary, for example listing off dates of different nuclear power plant developments. 

 - I think more background on what deep geological disposal is in the introduction would be helpful. 

 - The IAEA is already working heavily on plastic pollution, I don't know if it is necessary to have a new agency. 

 - What is "the cost of plastic ecology"?

 - The case for deep geological disposal seems like a very similar strategy as landfilling and this is not discussed in the current manuscript, why not just landfill plastic? It is clear to me why you would want to separate radioactive waste into its own long term management stream. 

 - Funding strategy seems convoluted, on one side it seems like the author is advocating for plastic to remain in circulation and have higher value, on the other it advocates for disposal and containment. 

 Good please keep:

 - I do agree with the authors that this is a new concept worth discussing and theorizing about.

Is the topic of the opinion article discussed accurately in the context of the current literature?

Partly

Are arguments sufficiently supported by evidence from the published literature?

Partly

Are all factual statements correct and adequately supported by citations?

Partly

Are the conclusions drawn balanced and justified on the basis of the presented arguments?

Partly

Reviewer Expertise:

Waste management, plastic pollution, watershed fate and transport

I confirm that I have read this submission and believe that I have an appropriate level of expertise to state that I do not consider it to be of an acceptable scientific standard, for reasons outlined above.

## References

[1] : A growing plastic smog, now estimated to be over 170 trillion plastic particles afloat in the world’s oceans—Urgent solutions required. *PLOS ONE* .2023;18(3) : 10.1371/journal.pone.0281596 10.1371/journal.pone.0281596 PMC999474236888681

[2] : Global mass of buoyant marine plastics dominated by large long-lived debris. *Nature Geoscience* .2023;16(8) : 10.1038/s41561-023-01216-0 689-694 10.1038/s41561-023-01216-0

# Response

**Published:** 2025-07-06

**Authors:** Hayk Minasyan

**Affiliations:** Biology, Yerevan State University, Yerevan, Yerevan, Armenia

Dear Dr. Win Cowger,

 Thank you very much for your valuable comments. I have revised the manuscript according to them. Below is my response to your criticism and suggestions.

 -Reviewer: Abstract not standard format, split into one two sentence paragraph and one longer one.

**Response**: Thank you for pointing this out. I have re-written the abstract.

 - Reviewer: "70% of plastic is polluting the environment" seems high, papers cited are not primary literature for that number. I recommend looking into the original literature those numbers came from and determine if that is accurate and cite the original source.

**Response**: I have replaced the data by the results of 2025 study (primary literature (Houssini et al., 2025).

 - Reviewer: This sentence is confusing:  "For example, it forms The Great Pacific Garbage Patch, which quickly captures new water surfaces ( Egger et al., 2020)."

**Response**: I have removed that sentence. It is not critical for presenting the concept of DGD.

 - Reviewer:  Many plastics in introduction just say there are large amounts of plastic, I think as a scientific study there should be numbers specifically stated, not just large amounts. For example, there are sources you can cite for how much plastic is in the ocean surface. I have added a few. 

**Response**: I agree, that precise numbers should be stated. Special thanks for your references: I have included their data to the article (Eriksen et al., 2023; Kaandorp et al.; 2023)

 - Reviewer: The first two sentences in the discussion could be combined.

**Response**: Agree. I have combined the first  two sentences in the discussion.

 - Reviewer: Unclear in text what the author means by plastic disintegration, do they mean leachates, microplastics, nanoplastics?

**Response**: I have revised the text for more concrete explanation of the meaning of plastic disintegration.

 - Reviewer: Some of the first paragraph of the discussion seems unnecessary, for example listing off dates of different nuclear power plant developments.

**Response**: I agree with you. I have removed the listing of different nuclear power plant developments from the text.

 - Reviewer: I think more background on what deep geological disposal is in the introduction would be helpful.

**Response**: Thank you for your suggestion. I have added more background on what deep geological disposal is in the introduction.

 - Reviewer: The IAEA is already working heavily on plastic pollution, I don't know if it is necessary to have a new agency.

**Response**: I have removed the fragment of the article regarding IAEA

 - Reviewer: What is "the cost of plastic ecology"?

**Response**: Thank you for your question. This concept encompasses both: a). **Direct economic costs**: cleanup, waste infrastructure, lost tourism and fishing revenue, health care due to contamination, infrastructure damage (e.g., flooding); b). **Ecological costs**: pollution of marine and terrestrial ecosystems, greenhouse emissions, biodiversity losses, chemicals leaching, damage to ecosystem services. The discussion of “the cost of plastic ecology” is not indispensable to   understand DGD concept and I have removed it from the text.

 References:

 1. Cordier, Mateo, Takuro Uehara, Bethany Jorgensen, and Juan Baztan. “Reducing Plastic Production: Economic Loss or Environmental Gain?” Cambridge Prisms: Plastics 2 (2024): e2. https://doi.org/10.1017/plc.2024.3.

 2. “Lifetime Cost of Plastic 10 Times Higher for Low-Income Countries than Rich Ones, Revealing Cri.” Accessed July 1, 2025. https://wwf.panda.org/wwf_news/?10004441/lifetime-cost-plastic

 3. “The Cost of Preventing Ocean Plastic Pollution.” OECD Environment Working Papers, March 4, 2022. https://doi.org/10.1787/5c41963b-en.

 4. Beaumont, Nicola J., Margrethe Aanesen, Melanie C. Austen, Tobias Börger, James R. Clark, Matthew Cole, Tara Hooper, Penelope K. Lindeque, Christine Pascoe, and Kayleigh J. Wyles. “Global Ecological, Social and Economic Impacts of Marine Plastic.” Marine Pollution Bulletin 142 (May 2019): 189–95. https://doi.org/10.1016/j.marpolbul.2019.03.022

 - Reviewer: The case for deep geological disposal seems like a very similar strategy as landfilling and this is not discussed in the current manuscript, why not just landfill plastic? It is clear to me why you would want to separate radioactive waste into its own long term management stream.

**Response**: Following your critical comment, I discussed and showed in the last paragraph of the Introduction the differences and numerous advantages of DGD over landfilling.

 - Reviewer: Funding strategy seems convoluted, on one side it seems like the author is advocating for plastic to remain in circulation and have higher value, on the other it advocates for disposal and containment.

**Response**: The funding concerns plastic waste and is aimed at stimulating its selective collection for further GDG. I have added explanations in the relevant parts of the text for greater clarity.

 Thank you again for your comments and suggestions

 Sincerely

 Hayk Minasyan

# Reviewer report: Reviewer response for version 1

**DOI:** 10.5256/f1000research.164194.r319698

**Published:** 2024-09-10

**Authors:** Magdalena Vaverková

**Affiliations:** 1 Warsaw University of Life Sciences, Warsaw, Poland

As someone who has been engaged in waste management for over 20 years, I found the article’s premise—deep geological disposal of plastic waste—intriguing, yet deeply problematic. While the article proposes this as a new approach to managing the vast quantities of mismanaged plastic waste (MPW), it overlooks several crucial aspects of plastic waste management that have proven to be more practical, efficient, and sustainable. I believe this concept lacks feasibility both from an economic and environmental standpoint.

 One of the most striking issues with the article is the inconsistency in its discussion of plastic’s chemical nature. On one hand, it is acknowledged that plastic can take hundreds to thousands of years to decompose, suggesting that it is inert and resistant to degradation. On the other hand, the article raises concerns about leachates and environmental contamination, which implies that plastic waste does, in fact, react and break down into harmful substances. These contradictions undermine the argument for deep geological disposal. If plastic is inert and non-reactive, the environmental risk should be minimal. However, if plastics release harmful chemicals, placing them in deep geological layers could lead to unforeseen contamination, much like the challenges associated with hazardous waste disposal.

 The suggestion to treat plastic waste similarly to nuclear waste by storing it in deep geological repositories overlooks the fact that plastic waste is far more abundant, widely dispersed, and less hazardous than nuclear waste. The costs associated with collecting, transporting, and storing plastic in deep geological repositories would be astronomical, and these financial burdens far outweigh any potential benefits. Furthermore, nuclear waste is stored this way due to its high-level, long-term radioactive danger. Plastic waste, while a significant pollutant, can be more effectively managed through other methods, including recycling, incineration for energy recovery, and the development of biodegradable alternatives.

 Over the years, I have seen substantial progress in both recycling technologies and waste-to-energy systems. Many types of plastic waste, particularly PET, can be effectively recycled, and non-recyclable plastics can be incinerated in highly regulated incinerators to produce energy. Japan’s approach to waste management, for example, includes advanced waste-to-energy systems that capture a significant amount of the country’s energy from non-recyclable waste while keeping emissions under tight control.

 By contrast, the article’s deep geological disposal concept ignores the benefits of energy recovery from plastic incineration. Given the enormous global need for energy and the ongoing development of incineration technologies that meet modern environmental standards, it would make much more sense to focus on optimizing energy recovery from plastic waste than to bury it in geological sites. Additionally, improving recycling infrastructure and reducing single-use plastics are more viable long-term strategies than deep geological disposal.

 The article does highlight one concept worth exploring further—treating plastic waste as a valuable commodity. However, rather than applying this idea to deep geological storage, it should be linked to the principles of a circular economy. In recent years, there has been growing interest in treating waste as a resource, and plastics are no exception. By developing better market mechanisms for plastic waste, particularly through innovations in chemical recycling or even upcycling processes, we could unlock economic opportunities while addressing the environmental challenges posed by plastic pollution.

 Conclusion

 While the article raises important concerns about the growing problem of plastic waste, the proposed solution of deep geological disposal is neither economically nor environmentally viable. Decades of experience in waste management have shown that more practical solutions—such as advanced recycling, waste-to-energy, and policy-driven reductions in plastic production—are far more promising. Furthermore, there is significant potential to treat plastic waste as a resource within a circular economy framework, which could help mitigate the environmental crisis caused by plastic waste without resorting to drastic, impractical measures like deep geological disposal.

 In my professional view, the future of plastic waste management lies in innovation, sustainable technologies, and responsible consumption, not in burying the problem underground.

Is the topic of the opinion article discussed accurately in the context of the current literature?

Yes

Are arguments sufficiently supported by evidence from the published literature?

No

Are all factual statements correct and adequately supported by citations?

No

Are the conclusions drawn balanced and justified on the basis of the presented arguments?

No

Reviewer Expertise:

Waste management

I confirm that I have read this submission and believe that I have an appropriate level of expertise to state that I do not consider it to be of an acceptable scientific standard, for reasons outlined above.

# Response

**Published:** 2024-09-11

**Authors:** Hayk Minasyan

**Affiliations:** Biology, Yerevan State University, Yerevan, Yerevan, Armenia

Article

**Deep geological disposal of plastic waste: Pros and cons**

**Hayk Minasyan**

 Hayk Minasyna’s response to the review of  Magdalena Vaverková

 Author Response to Reviewer

Author response:

 Dear  Magdalena Vaverková,

 Warsaw University of Life Sciences, Warsaw, Poland 

  Thank you for giving me the opportunity to start a discussion with you regarding your critical comments. I wrote at the end of my article: “This approach to plastic waste management requires future research and discussion regarding the optimization of the concept, its practices, regulations, and policies.” Unfortunately, you did not come up with any specific rational proposal, analysis of the concept, or attempt to optimize the concept or use its elements that, in your opinion, could be useful.

 However, any radically negative response can be useful in igniting debate and bringing the idea of ​​deep geological disposal of plastic waste to the attention of open-minded researchers. I will try to provide references, where possible, from my article, so that they are easier to find.

Reviewer comments

 As someone who has been engaged in waste management for over 20 years, I found the article’s premise—deep geological disposal of plastic waste—intriguing, yet deeply problematic.

Author response: In the last 20 years, the amount of plastic waste has more than **doubled** (OECD Global Plastics Outlook: Policy Scenarios to 2060 OECD Publishing (2022)

https://www.oecd-ilibrary.org/environment/global-plastics-outlook_aa1edf33-en ]

 It means that a solution to the problem has not yet been found.

Reviewer comments

  While the article proposes this as a new approach to managing the vast quantities of mismanaged plastic waste (MPW), it overlooks several crucial aspects of plastic waste management that have proven to be more practical, efficient, and sustainable. I believe this concept lacks feasibility both from an economic and environmental standpoint.

Author response: While belief holds great value in many discussions, it is not typically considered a scientific measure. In order to assess the feasibility of the concept from both economic and environmental perspectives, it would be helpful if the reviewer could present relevant economic calculations, scientific data, and detailed explanations.

Reviewer comments

 One of the most striking issues with the article is the inconsistency in its discussion of plastic’s chemical nature. On one hand, it is acknowledged that plastic can take hundreds to thousands of years to decompose, suggesting that it is inert and resistant to degradation. On the other hand, the article raises concerns about leachates and environmental contamination, which implies that plastic waste does react and break down into harmful substances.

Author response: There is no inconsistency:  plastic waste can generate leachate, a liquid that forms when water **percolates through waste. **In deep geological disposal with appropriate technical conditions (isolation, sealing walls for liquids, etc.) plastic waste **breakdown is excluded** and **leachate generation is impossible**.

Reviewer comments

 These contradictions undermine the argument for deep geological disposal.

Author response: There are no contradictions. In a duly technical approach, nothing undermines the idea of deep geological disposal.

Reviewer comments

  If plastic is inert and non-reactive, the environmental risk should be minimal. However, if plastics release harmful chemicals, placing them in deep geological layers could lead to unforeseen contamination, much like the challenges associated with hazardous waste disposal.

 .

Author response: Disposal of plastic waste in deep and dry repositories is **safe and can’t lead to contamination.**

Reviewer comments

 The suggestion to treat plastic waste similarly to nuclear waste by storing it in deep geological repositories overlooks the fact that plastic waste is far more abundant, widely dispersed, and less hazardous than nuclear waste.

Author response: Yes, plastic waste is less hazardous and more abundant than nuclear waste, but this argument is not something principally against deep geological disposal of plastic waste.

Reviewer comments

 The costs associated with collecting, transporting, and storing plastic in deep geological repositories would be astronomical, and these financial burdens far outweigh any potential benefits.

Author response: To make such assertions with confidence, it would also be expected that the reviewer possess a high level of expertise in the field of economics, supported by financial calculations and thorough estimates. Moreover, the reviewer appears to overlook the current limitations of methods like recycling, incineration for energy recovery, and the development of biodegradable alternatives. These approaches, while promising, have yet to demonstrate sufficient effectiveness to curb the rapid accumulation of plastic waste.

Reviewer comments

 Furthermore, nuclear waste is stored this way due to its top-level, long-term radioactive danger.

Author response: Yes, nuclear waste is stored this way due to its high-level, long-term radioactive danger, but again, it does not mean that plastic waste may not be stored by deep geological technology.

Reviewer comments

  Plastic waste, while a significant pollutant, can be more effectively managed through other methods, including recycling, incineration for energy recovery, and the development of biodegradable alternatives.

Author response: The reviewer ignores the fact that the methods she mentions, including recycling, incineration for energy recovery, and the development of biodegradable alternatives, are not yet effective enough and plastic waste continues to accumulate rapidly.

 I would like to draw the reviewer's attention to the fact that no more than 10% of plastic waste has been recycled, 11% burned, 8% buried, and the remaining 70% pollutes the environment ( Enfrin et al., 2019 , Zaman and Newman, 2021 ).

Reviewer comments

 Over the years, I have seen substantial progress in both recycling technologies and waste-to-energy systems.

Author response: Nevertheless, at present recycling technology processes only 10% of plastic waste; waste-to-energy technology burns 11% of plastic waste.

Reviewer comments

  Many types of plastic waste, particularly PET, can be effectively recycled, and non-recyclable plastics can be incinerated in highly regulated incinerators to produce energy.

Author response: While recycling is an important tool, it cannot be viewed as a standalone solution to the plastic waste crisis. The complex nature of the problem requires more than one method of management.

 By the year 2060, there is a projected trajectory for plastic waste to experience an approximately threefold increase. Within this context, nearly 50 % of the total plastic waste will continue to be deposited in landfills, **with a recycling rate of <20 %** (OECD Global Plastics Outlook: Policy Scenarios to 2060 OECD Publishing (2022) https://www.oecd-ilibrary.org/environment/global-plastics-outlook_aa1edf33-en ).

 In addition, recycling plastic is not preferable in most cases because it is technologically complex and expensive, many types of plastic cannot be reused, and finally, it is impossible to recycle the same plastic endlessly.

References:

 Aydin M, Degirmenci T, Bozatli O, Balsalobre-Lorente D. Fresh evidence of the impact of economic complexity, health expenditure, natural resources, plastic consumption, and renewable energy in air pollution deaths in the USA? An empirical approach. Science of The Total Environment [Internet]. 2024 Apr;921:171127. Available from: https://linkinghub.elsevier.com/retrieve/pii/S004896972401266X )

 Dong L, Huang Z, Qin Y, Zhi W. Industrialized fine physical regeneration process and economic benefit assessment for recycling waste HDPE containers. Journal of Cleaner Production [Internet]. 2024 Apr [cited 2024 Sep 10];447:141477. Available from: https://linkinghub.elsevier.com/retrieve/pii/S0959652624009259

 Merrington A. Recycling of plastics. In: Applied Plastics Engineering Handbook [Internet]. Elsevier; 2024 [cited 2024 Sep 10]. p. 191–217. Available from: https://linkinghub.elsevier.com/retrieve/pii/B9780323886673000060

Reviewer comments

 Japan’s approach to waste management, for example, includes advanced waste-to-energy systems that capture a significant amount of the country’s energy from non-recyclable waste while keeping emissions under tight control.

 By contrast, the article’s deep geological disposal concept ignores the benefits of energy recovery from plastic incineration.

Author response: It seems there may have been some misunderstanding or misinterpretation of my article. The reviewer contrasts the ideas presented in the article with current waste management technologies without offering a clear logical basis for such conclusions. This leads to certain assumptions that remain unproven. The idea of deep geological disposal of plastic waste does not ignore other technologies of waste management including plastic incineration. It should be used simultaneously with other technologies.  In combination with all modern technologies of plastic waste management, deep geological disposal may help to mitigate the problem of plastic waste.

 As to plastic incineration, it is dangerous for the environment due to ecological risks:

1. **Air Pollution**: Incineration releases toxic heavy metals, dioxins, furans, into the air. They poison humans and animals. Dioxins and furans accumulate in the food chain.
2. **Greenhouse Gas Emissions**: Burning plastic releases carbon dioxide (CO₂) and other greenhouse gases, contributing to climate change.
3. **Toxic Ash**: Incineration generates toxic ash that needs to be disposed of in landfills and can contaminate soil and water.
4. **Microplastics**: Incineration may produce microplastics that can be released into the atmosphere or water systems, causing further ecological harm.

Due to these risks, incineration is not a preferred method for managing plastic waste.

References:

 Luo L, Guo S, Shen D, Shentu J, Lu L, Qi S, et al. Characteristics and release potential of microplastics in municipal solid waste incineration bottom ash. Chemosphere [Internet]. 2024 Sep [cited 2024 Sep 10];364:143163. Available from: https://linkinghub.elsevier.com/retrieve/pii/S0045653524020605

 Jin M, Sun M, Liu J, Dong C, Xue J. Influence of operating parameters on the yield of micro-plastics from plastics incineration. Science of The Total Environment [Internet]. 2024 Feb [cited 2024 Sep 10];912:169347. Available from: https://linkinghub.elsevier.com/retrieve/pii/S0048969723079779

 Rogers K, WaMaina E, Barber A, Masood S, Love C, Kim YH, et al. Emissions from plastic incineration induce inflammation, oxidative stress, and impaired bioenergetics in primary human respiratory epithelial cells. Toxicological Sciences [Internet]. 2024 May 28 [cited 2024 Sep 10];199(2):301–15. Available from: https://academic.oup.com/toxsci/article/199/2/301/7636471

Reviewer comments

 Given the enormous global need for energy and the ongoing development of incineration technologies that meet modern environmental standards, it would make much more sense to focus on optimizing energy recovery from plastic waste than to bury it in geological sites.

Author response:  The reviewer strongly overestimates plastic incineration.  At present, there are no incineration technologies that meet modern environmental standards:

References:

 Jung S, Jung H, Ahn Y. Plastic-to-energy: Process and economic–environmental assessment of a recycling technology. Process Safety and Environmental Protection [Internet]. 2024 Mar [cited 2024 Sep 10];183:1051–8. Available from: https://linkinghub.elsevier.com/retrieve/pii/S0957582024000806

 Pathak G, Nichter M, Hardon A, Moyer E. The open burning of plastic wastes is an urgent global health issue. Annals of Global Health [Internet]. 2024 Jan 12 [cited 2024 Sep 10];90(1):3. Available from: https://annalsofglobalhealth.org/articles/10.5334/aogh.4232/

 Besides, energy recovery from plastic waste (plastic incineration) results in irreversible loss of plastic. Burying plastic in the ground preserves plastic for future generations of people when the planet's hydrocarbons run out. As for energy production, from the ecological point of view, it must be obtained from renewable sources such as solar energy, wind, hydropower, etc. In this sense, nuclear energy is much more efficient and ecologically safer than burning plastic.

References:

 Pourasl HH, Barenji RV, Khojastehnezhad VM. Solar energy status in the world: A comprehensive review. Energy Reports [Internet]. 2023 Nov [cited 2024 Sep 11];10:3474–93. Available from: https://linkinghub.elsevier.com/retrieve/pii/S2352484723014579

 Kaldellis JK, Boulogiorgou D. Renewable energy: Wind energy. In: Living with Climate Change [Internet]. Elsevier; 2024 [cited 2024 Sep 11]. p. 513–57. Available from: https://linkinghub.elsevier.com/retrieve/pii/B9780443185151000174

 Hatamkhani A, Moridi A, Haghighi AT. Incorporating ecosystem services value into the optimal development of hydropower projects. Renewable Energy [Internet]. 2023 Feb [cited 2024 Sep 11];203:495–505. Available from: https://linkinghub.elsevier.com/retrieve/pii/S0960148122018754

 Krūmiņš J, Kļaviņš M. Investigating the potential of nuclear energy in achieving a carbon-free energy future. Energies [Internet]. 2023 Apr 22 [cited 2024 Sep 11];16(9):3612. Available from: https://www.mdpi.com/1996-1073/16/9/3612

Reviewer comments

 Additionally, improving recycling infrastructure and reducing single-use plastics are more viable long-term strategies than deep geological disposal.

Author response: The reviewer appears to contrast the ideas presented in the article with existing plastic waste management technologies without providing clear justification for these comparisons, which seems to lead to conclusions that may not be fully substantiated.

Reviewer comments

 The article does highlight one concept worth exploring further—treating plastic waste as a valuable commodity. However, rather than applying this idea to deep geological storage, it should be linked to the principles of a circular economy.

Author response: Plastic is a valuable commodity and it is true for both deep geological storage and the principles of a circular economy. It is true for all other technologies as well.  The reviewer persistently contrasts deep geological disposal versus the principles of a circular economy.  Applying the idea of plastic waste's monetary value to deep geological storage does not exclude its application to the principles of a circular economy and other strategies.

Reviewer comments

  In recent years, there has been growing interest in treating waste as a resource, and plastics are no exception. By developing better market mechanisms for plastic waste, particularly through innovations in chemical recycling or even upcycling processes, we could unlock economic opportunities while addressing the environmental challenges posed by plastic pollution.

Author response: It sounds like a quote from a public speech. Yes, in recent years there has been growing interest in treating waste as a resource. However, plastic waste continues to accumulate on land, seas, and oceans.

 Reviewer's Conclusion

Reviewer comments

 While the article raises important concerns about the growing problem of plastic waste, the proposed solution of deep geological disposal is neither economically nor environmentally viable.

Author response: Again, for such a categorical conclusion the reviewer should present economic calculations and reasonable explanations as to why deep geological disposal is not environmentally viable.

Reviewer comments

 Decades of experience in waste management have shown that more practical solutions—such as advanced recycling, waste-to-energy, and policy-driven reductions in plastic production—are far more promising.

Author response: Maybe the solutions mentioned are promising, but today plastic waste continues accumulating. Again the same statistics:  10% of waste has been recycled, 11% burned, 8% buried, and the remaining 70% pollutes the environment. Declarative statements about promising technologies do not change the catastrophic state of affairs with waste accumulation. My article proposes additional measures to collect plastic from the seas, oceans, and landfills and bury it safely as a resource for future generations. Deep geological disposal is not opposed to all other plastic waste management technologies, but is offered as an additional tool to solve together with other technologies the very serious problems facing humanity in connection with plastic waste.

Reviewer comments

 Furthermore, there is significant potential to treat plastic waste as a resource within a circular economy framework, which could help mitigate the environmental crisis caused by plastic waste without resorting to drastic, impractical measures like deep geological disposal.

 In my professional view, the future of plastic waste management lies in innovation, sustainable technologies, and responsible consumption, not in burying the problem underground.

Author response: Тhe reviewer frames her perspective as a personal professional stance, though it aligns with the widely accepted principles of innovation, sustainable technologies, and responsible consumption in waste management. Regarding the suggestion of burying the problem underground, the article specifically advocates for the storage of plastic waste that cannot be recycled, has already undergone multiple recycling processes, or poses significant risks if incinerated or processed by existing technologies.

 Unfortunately, the primary concern raised by Dr. Magdalena Vaverková centers on a rather dismissive critique of deep geological disposal, which seems to overshadow other important aspects of the article. Her stance appears to impede an objective review of other proposed ideas, such as mechanisms for collecting plastic waste from oceans, seas, and landfills, as well as the organizational, political, and financial strategies for implementing deep geological disposal.
